# Open-ended urethral catheters reduce catheter obstruction after hypospadias repair

**DOI:** 10.3389/fped.2024.1402440

**Published:** 2024-12-19

**Authors:** Xiang Zhao, Kechi Yu, Erhu Fang, Ning Li

**Affiliations:** Department of Pediatric Surgery, Tongji Hospital, Tongji Medical College, Huazhong University of Science and Technology, Wuhan, China

**Keywords:** open-ended, urethral catheter, catheter obstruction, hypospadias, pediatric

## Abstract

**Backgrounds:**

Urethral catheter obstruction is a notable issue that pediatric patients with hypospadias may encounter in the early postoperative period. This retrospective study aims to assess the efficacy of open-ended urethral catheters with 2 side holes in mitigating catheter obstruction in pediatric patients following hypospadias repair.

**Materials and methods:**

The clinical data of pediatric patients who underwent hypospadias repair surgery from January 2021 to October 2023 were retrospectively collected. The patients were divided into 2 groups. Those who used standard Foley catheters were referred to as Group A, while those who used modified open-ended catheters were referred to as Group B. The primary outcome was the incidence of catheter obstruction within 7 days postoperatively.

**Results:**

A total of 297 patients were included in this study, with 142 patients in Group A and 155 patients in Group B. In Group A, there were 12 cases of catheter obstruction, with 10 cases resolved through irrigation and 2 cases requiring additional catheters insertion by suprapubic bladder punctures. In Group B, only 4 cases experienced catheter obstruction, which were effectively and easily resolved through maneuver irrigation. The incidence of catheter obstruction in Group B was statistically significantly lower than that in Group A (2.6% vs. 8.5%, *p* < 0.05).

**Conclusion:**

In pediatric hypospadias patients who underwent surgical repair, the use of open-ended urethral catheters with 2 side holes significantly diminishes the incidence of postoperative catheter obstruction. This simple technique is worthy of promotion.

## 1 Introduction

The placement of urethral catheters or stents is generally a routine procedure in hypospadias repair. Some surgeons prefer to use urethral stent which is secured at the glans, while others are accustomed to using Foley catheters, and the duration of the postoperative indwelling time varies among different surgeons (1–3). In our institution, all-silicone Foley catheters are typically retained for a duration of 7–14 days postoperatively in patients who have undergone hypospadias repair.

Indwelling urethral catheters may lead to various issues, including urinary tract infection, urethral or meatal trauma, bladder spasms, and catheter blockage, with catheter blockage posing a significant challenge for patients who have undergone hypospadias repair (2, 4–6). Smaller catheters sizes are more susceptible to experiencing blockage situations. However, catheters with larger size may exert pressure on surrounding tissues, thus increasing the risk of urethral stenosis (7). In hypospadias repair for pediatric patients, the most commonly used urethral catheters sizes are 6 Fr or 8 Fr, with size 6 Fr being the predominant choice in our institution. However, size 6 Fr catheters are more susceptible to experiencing blockage.

Preventing urethral catheter obstruction is an important aspect of postoperative management for hypospadias patients. Ensuring an adequate volume of urinary output can reduce the risk of urethral catheter obstruction, but blockage situations can still occur, posing challenges for postoperative management and causing physical discomfort in patients. The classic Foley catheter is designed with a closed-ended tip, making it less amenable to irrigation for clearance in the event of blockage. During irrigation, blood clots or tissue debris may become lodged at the closed tip, making clearance difficult.

One study reported that the larger the total area of all drainage holes in the urinary catheter, the easier it is to be irrigated for clearance (8). At first, in order to facilitate easier irrigation in the event of blockage, we excised the closed tip of the urinary catheter but retained its 2 side holes. Surprisingly, it was observed that this open-ended configuration significantly reduced the occurrence rate of catheter blockage. In this retrospective study, we aim to evaluate the effectiveness of open-ended urethral catheters with 2 lateral holes in mitigating catheter obstruction in pediatric patients following hypospadias repair.

## 2 Materials and methods

### 2.1 Patients and study design

The present study was designed retrospectively. The medical records of pediatric patients who have undergone hypospadias repair surgery from January 2021 to October 2023 were retrospectively reviewed. Only those patients utilized 6 Fr urethral catheters were enrolled in this study. Most of the pediatric patients received hypospadias repair used 6 Fr catheters in our institution. Patients with older age or those received staged surgeries usually utilized catheters of size 8 Fr or larger, and as such, were excluded from this study. All patients received prophylactic intravenous antibiotics once on the day of the surgery. Patients were encouraged to increase their fluid intake beginning on the second day post-surgery to ensure an adequate urinary output.

In our country, the tiered medical system is not yet adequately developed. If patients are discharged immediately after hypospadias surgery, they won't receive adequate postoperative care. Thus, in our institution, patients typically remain hospitalized for a minimum of 7 days postoperatively. The data about the occurrence of urinary catheter obstruction within the first postoperative 7 days was collected. Other clinical data includes patients age, hypospadias types, surgical techniques, presence of gross hematuria, and occurrence of urethral fistula were also collected. All patients were followed up for a minimum of 6 months to ascertain the occurrence of urethral fistula.

The patients were categorized into 2 groups: Group A comprised patients who used standard Foley catheters with closed end and 2 side holes, whereas Group B included those who used modified open-ended catheters with 2 side holes. Since August 2022, we have introduced the modified open-ended catheters, replacing the conventional closed-end Foley catheters that were previous employed.

The present study was reviewed and approved by the local medical ethics committee. The research was conducted in accordance with the principles outlined in the Declaration of Helsinki. Informed consent was exempted due to the retrospective design.

### 2.2 Technique

**Figure 1:**
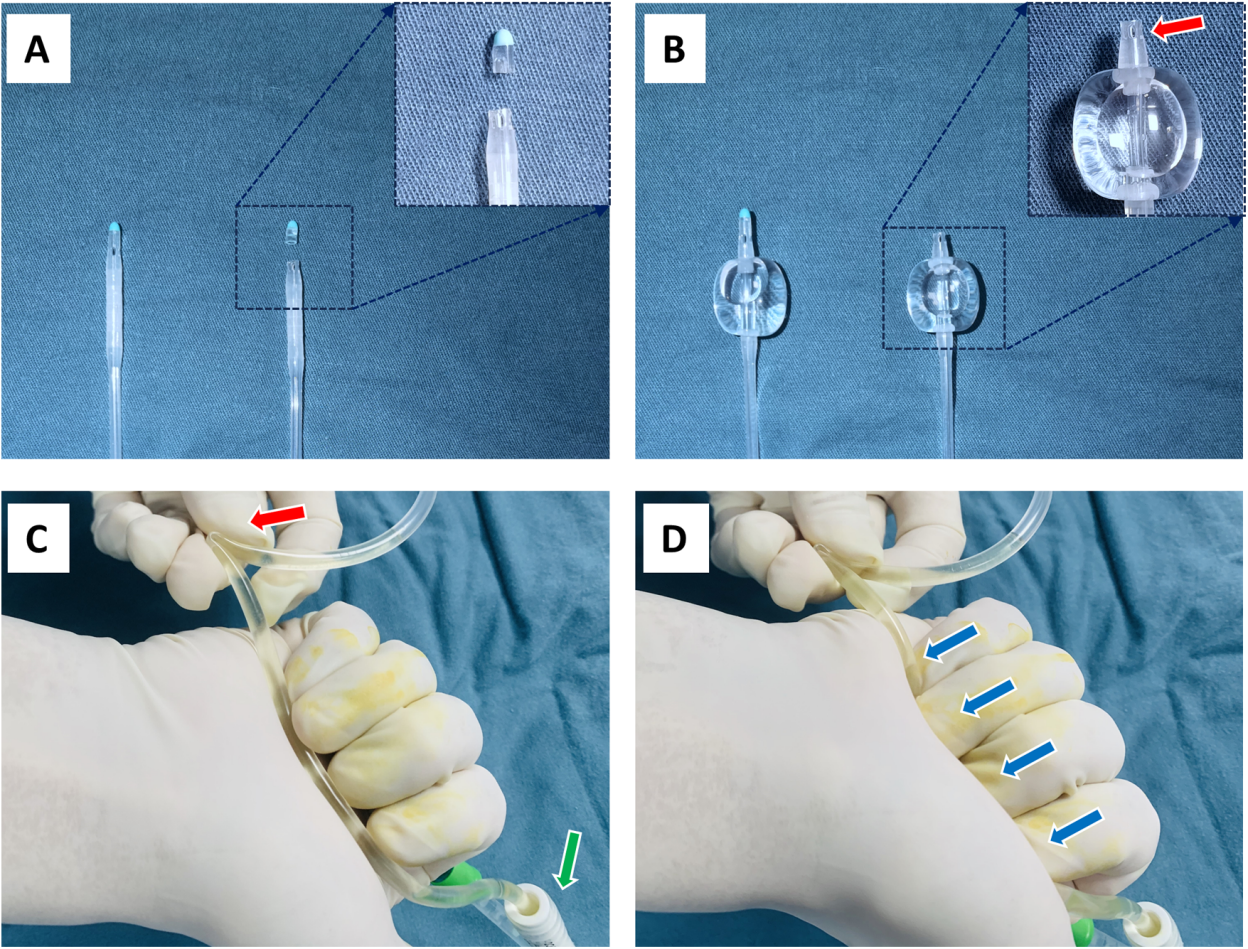
**(A)** The closed-end of the conventional urinary catheter was excised. The left is the conventional Foley catheter, the right is the modified open-ended catheter. **(B)** The modified open-ended catheter has a low-profile with shorter tail. The 2 lateral holes were preserved (red arrow). **(C)** Fold and secure the urinary bag tube approximately 15 cm away from the urinary catheter (red arrow). The green arrow indicates the outside end of the urinary catheter. **(D)** Grip the proximal segment of the urinary bag tube with the palm and four fingertips, repeatedly applying pressure to irrigate the urinary catheter (blue arrow).

All the catheters used in our institution are made of silicon. The conventional Foley urethral catheters are closed-ended and have 2 lateral drainage holes. The modified open-ended catheters were developed by excising the sealed ends of the conventional Foley catheters (Figure 1A), while retaining the 2 side holes of the catheter, thus yielding a total of 3 drainage openings (Figure 1B). The modified open-ended catheter has a low-profile with shorter tail (Figure 1B). All the catheters were transurethrally placed during the surgical procedure of hypospadias repair.

When encountering a urinary catheter obstruction, we typically follow a two-step procedure for clearance. The first step is a simple irrigation maneuver: fold and fasten the tube of the urinary drainage bag approximately 15 cm away from the urinary catheter (Figure 1C), then grip the proximal segment of the urinary bag tube with the palm and four fingertips, repeatedly applying pressure several times to irrigate the urinary catheter (Figure 1D). In cases of mild catheter obstruction, this maneuver can effectively relieve the blockage. If the first-step maneuver fails to clear the obstruction, the second step irrigation is initiated: use 50 ml syringe (pre-loaded with about 20 ml normal saline solution) to repeatedly irrigate the urinary catheter, aiming to agitate the clots or debris.

### 2.3 Statistical analysis

Chi-square test or Chi-squared test with Yates' correction was employed to compare categorical variables across distinct groups, while Student's *t*-test was utilized for the comparison of continuous variables. Statistical analyses were conducted using R software (version 4.2.2), with a statistical significance level set at *p* < 0.05.

## 3 Results

A total of 297 pediatric patients were included in this study, with 142 cases using conventional closed-ended Foley catheters (group A) and 155 cases using modified open-ended catheters (group B). Removing the closed end does not increase the difficulty of inserting the urinary catheter. All the modified open-ended catheters were successfully placed in Group B patients during the surgery. The clinical characteristics of participants were comparable across the groups (Table 1).

**Table 1:** Clinical characteristics and outcomes for the two groups.

Patients	Group A	Group B	*p*-value
Total cases	142	155	–
Mean age, months	17.1 ± 7.3	18.4 ± 7.1	0.13
Surgery technique			0.87
Mathieu	5 (3.5%)	5 (3.2%)	
Tubularized incised plate (TIP)	112 (78.9%)	126 (81.3%)	
Duckett or Koyanagi	25 (17.6%)	24 (15.5%)	
Catheter blockage	12 (8.5%)	4 (2.6%)	0.048*
Gross hematuria	11 (7.7%)	6 (3.9%)	0.15
Urethral fistula	13 (9.2%)	11 (7.1%)	0.52

* Chi-squared test with Yates’ correction.

A total of 12 patients in Group A experienced urinary catheter obstruction, whereas only 4 patients in Group B encountered this issue (Table 1). The difference of the incidences of catheter obstruction between the two groups is statistically significant (8.5% vs. 2.6%, *p* = 0.048).

The urinary catheter obstruction in all the 4 cases from Group B was resolved by using the first-step simple maneuver, whereas only 2 cases from Group A was resolved by repeating the first-step maneuver. The catheter obstruction in 8 cases from Group A was resolved through repeated saline irrigation. In 2 cases from Group A, the catheter blockage cannot be resolved by either the first-step maneuver or saline irrigation. In these 2 cases the blockage occurred too early postoperatively (the second day and third day after surgery, respectively), making it very difficult to replace the catheters. Therefore, suprapubic bladder punctures were performed to place additional drainage catheters for these 2 cases, while the blocked catheters were retained as supporting tubes for the newly created urethras.

Gross hematuria refers to the presence of visible blood in the urine, which can manifest as a pink, red, or brownish hue. It usually occurs 1–2 days after surgery and continues until the catheter is removed. Gross hematuria was observed in 11 cases from Group A and in only 6 cases from Group B; however, the difference was not statistically significant. The relative low-profile (shorter tail) of the open-ended catheters may decrease the damage of the bladder mucosa (9, 10), and thus may be responsible for the lower rate of gross hematuria.

Gross hematuria is related to urinary catheter blockage, because it can lead to blood clots. However, gross hematuria did not always lead to catheter obstruction. The urinary catheter obstruction in 7 cases from Group A and in 1 case from Group B were caused by obvious blood clots. In the other cases, the obstructions were caused by tissue debris or crystals.

There was no significant difference in the incidence of urethral fistula between the two groups (9.2% vs. 7.1%).

## 4 Discussion

Hypospadias is one of the most common congenital abnormalities in boys. It is characterized primarily by the urethral meatus not being located at the tip of the penis but instead being positioned along the ventral side of the penis, scrotum, or perineum (1, 11). In addition to the abnormal positioning of the meatus, hypospadias often involves varying degrees of chordee, incomplete foreskin, and hypoplasia of the ventral penile skin (1). The Hypospadias International Society (HIS) classified hypospadias into four categories based on the position of the displaced urethral meatus and degree of chordee: grade I or glanular hypospadias, grade II or distal hypospadias, grade III or proximal hypospadias, and grade IV or perineal hypospadias which is usually associated with severe chordee (12).

This developmental anomaly can only be corrected through surgical repair. It is reported that there are more than 250 surgery techniques for hypospadias repair (11). For glanular or distal hypospadias, the Mathieu technique, meatal advancement and glansplasty integrated (MAGPI) technique, and tubularized incised plate (TIP) technique are the most commonly used (1, 3, 13). For proximal or perineal hypospadias, TIP urethroplasty, Onlay island flap urethroplasty, Duckett urethroplasty, and Koyanagi urethroplasty are the most commonly used (1, 3, 14). For severe cases of perineal hypospadias, two-staged surgeries are often required. In more than 95% of the cases, a urethral catheter or stent is placed during the surgery and left in place for 3–7 days or more postoperatively (3). In our institution, most of the pediatric hypospadias patients received 6 Fr Foley catheter during the surgery and often retained for a duration of 7–14 days postoperatively.

Although the incidence of catheter obstruction is not very high for children who received hypospadias repair, it still poses challenges for postoperative management. Urinary catheter obstruction contributes to urine bypassing and brings more physical discomfort for the children. If the catheter obstruction cannot be resolved through washout, it is typically necessary to insert a new catheter, which bring more pain for the children. Moreover, if the urinary catheter obstruction occurs in the first few days postoperatively and cannot be resolved through washout, it is often very difficult to replace the catheter, thus necessitating a suprapubic bladder puncture for the placement of a new catheter.

Unfortunately, we found that, for the traditional closed-ended Foley catheters, once a urinary catheter obstruction occurs, it is often very hard to clear through washout. We speculated that this is due to blood clots or tissue debris becoming trapped in the closed-ended tip of the urinary catheter during the irrigation process. Therefore, we started to trim the sealed end of the Foley catheter tip, but preserving its 2 lateral holes, in the hope that it would facilitate easier clearance in the event of catheter obstruction. Surprisingly, we observed that this technique also reduced the incidence of catheter obstruction. In this study, the total rate of catheter blockage in the open-ended catheter group is significantly lower than that in the traditional closed-ended catheter group (2.6% vs. 8.5%, *p* = 0.048).

We believe that the following reasons may responsible for the reduced occurrence of urethral obstruction with the this modified open-ended urinary catheter.

Firstly, this modified open-ended catheter with 2 lateral holes increases the total drainage area of the openings. It is reasonable that increased number of drainage openings and a larger total area would result in a reduced likelihood of blockages.

Secondly, the relatively shorter tip of the urinary catheter makes it less likely to damage the bladder mucosa, thereby reducing the generation of blood clots and tissue debris. Two study reported that urethral catheters with open-ended designs and shorter tails (shorter distance from the balloon base to the catheter tip) could decrease the damage to the bladder mucosa (9, 10). In this study, although the difference is not statistically different, the rate of gross hematuria in the open-ended catheter group is lower than that in the traditional closed-ended catheter group (3.9% vs. 7.7%).

Thirdly, according to the findings from Farahnaz's study (10), low-profile urinary catheters with shorter tail reduces the incidence of catheter-associated urinary tract infections, consequently lowering the incidence of bacteria-related encrustation. As is well known, urinary tract infections and the presence of urease-producing bacteria can result in crystal formation within the urine, leading to encrustation in the catheter lumen and openings, ultimately causing the catheter to become blocked (4, 15, 16).

There are also some limitations for this study. Firstly, this is a retrospectively designed study, prospective randomized controlled trials are needed to further validate the conclusions. Secondly, this study only enrolled patients using 6 Fr catheters; therefore, the research findings may not necessarily be applicable to other sizes of urinary catheters. Thirdly, the urinary catheter retention duration varies among different surgeons and different types of hypospadias, so the findings of this study may not be suitable to cases where the indwelling catheter duration is less than 7 days. Fourthly, the reasons for reducing catheter obstruction mentioned above are speculated based on other studies and require more direct evidence for confirmation.

In conclusion, for pediatric hypospadias patients used 6 Fr urinary catheter during the repair surgery, open-ended catheters with 2 lateral holes could reduce the incidence of catheter blockage. These open-ended catheters can be easily developed by excising the closed end of the conventual Foley urinary catheter while retaining its 2 side holes.

## Data Availability

The original contributions presented in the study are included in the article/Supplementary Material, further inquiries can be directed to the corresponding author.
